# Identification and genetic analysis of a pervasive ‘needle-eye’ sperm phenotype in *Drosophila* sterile hybrid males

**DOI:** 10.1098/rspb.2024.0483

**Published:** 2024-06-19

**Authors:** Rachelle L. Kanippayoor, Charles Soeder, Tom Hsiang, Corbin D. Jones, Amanda J. Moehring

**Affiliations:** ^1^ Department of Biology, Western University, London, Ontario N6A 5B7, Canada; ^2^ Department of Biology, University of North Carolina at Chapel Hill, Chapel Hill, NC 27599, USA; ^3^ School of Environmental Sciences, University of Guelph, Guelph, Ontario N1G 2W1, Canada

**Keywords:** postzygotic isolation, hybrid dysfunction, *Drosophila simulans*, *Drosophila mauritiana*, *Drosophila pseudoobscura*, *Drosophila mojavensis*

## Abstract

Interspecies hybrid sterility has been extensively studied, especially in the genus *Drosophila*. Hybrid sterility is more often found in the heterogametic (XY or ZW) sex, a trend called Haldane’s rule. Although this phenomenon is pervasive, identification of a common genetic mechanism remains elusive, with modest support found for a range of potential theories. Here, we identify a single precise morphological phenotype, which we call ‘needle-eye sperm’, that is associated with hybrid sterility in three separate species pairs that span the *Drosophila* genus. The nature of the phenotype indicates a common point of meiotic failure in sterile hybrid males. We used 10 generations of backcross selection paired with whole-genome pooled sequencing to genetically map the regions underlying the needle-eye (NE) sperm phenotype. Surprisingly, the sterility phenotype was present in ~50% of males even after 10 generations of backcrossing, and only a single region of the X chromosome was associated with sterility in one direction of backcross. Owing to the common phenotype among sterile male hybrids*,* and the strong effect of individual loci, further exploration of these findings may identify a universal mechanism for the evolution of hybrid sterility.

## 1. Introduction

When two species interbreed, they often produce hybrids with fitness defects. Extreme hybrid dysfunction, such as sterility or inviability, can act as a barrier preventing gene flow between closely related species that otherwise could potentially interbreed and merge [1]. The prevailing model used to explain the mechanism underlying these dysfunctions is the Bateson–Dobzhansky–Muller (BDM) incompatibility model [2–4]. Populations evolving independently of each other can acquire and fix different mutations [5]. While these genomic changes are compatible within each population, they may be incompatible in the genetic background of the other population, which evolved along an independent trajectory, resulting in dysfunctional interpopulation hybrids (e.g. [6–8] but see [9]).

Hybrid sterility, an intrinsic postzygotic reproductive barrier, is present in many interspecific crosses (e.g. [1,6,8,10–12]). A notable trend in interspecies hybrids is that if only one sex is affected by postzygotic isolation, it is the heterogametic (e.g. XY or ZW) sex, a trend referred to as Haldane’s rule [13–15]. Since the pattern of heterogametic sterility is observed among almost all interspecies hybrids, a common underlying basis for the evolution of hybrid sterility may exist across multiple taxa.

Multiple theories have been developed to explain the presence of Haldane’s rule and hybrid sterility, including dominance theory, faster male, faster X or meiotic drive (reviewed in [1]). Each of these theories has some level of empirical support (e.g. [1,12,16–21]), with no single theory emerging as a universal explanation for Haldane’s rule and sterility [11]. However, among these theories, the dominance theory is most commonly evoked to explain Haldane’s rule. In this theory, a recessive sex-linked locus of one species has a negative interaction with a dominant autosomal-linked locus of another species, resulting in developmental defects that render the hybrid sterile [22–24]. As the sex-linked locus is recessive, heterogametic hybrids are disproportionately affected over homogametic hybrids, owing to the presence of a single copy of each sex chromosome in heterogametic hybrids. While there is strong support for the dominance model as a common mechanism underlying hybrid inviability [2–4,25], the surprising difficulty in identifying individual loci for F_1_ hybrid sterility has meant that, unlike inviability, there is limited support for the dominance model for sterility [11,17,18,26,27].

If the dominance theory is correct, random genetic changes that accumulate within a population would interact negatively when introduced into the genetic background of a different population. In *Drosophila*, we would expect males to be more susceptible to hybrid sterility than females if these random genetic incompatibilities were more commonly found on the X chromosome [28,29] or if spermatogenesis was overall more sensitive to genetic disruptions than other processes involved in development [21]. In both scenarios, however, random genetic mutations that lead to hybrid sterility in different species pairs are unlikely to affect the exact same point in spermatogenesis in the sterile hybrid produced by each pair, unless a single step of spermatogenesis is particularly sensitive to disruptive changes. Surprisingly, this may be the case as the precise spermatogenic failure phenotypes are remarkably similar among sterile interspecies hybrids across the *Drosophila* genus [30].

Here, we provide evidence that supports a consistent spermatogenic failure producing the same unusual sperm phenotype in hybrids from across the *Drosophila* genus. We focus on recently diverged taxa where sterile males still produce some sperm since genetic incompatibilities beyond those responsible for Haldane’s rule would continue to accumulate over the course of speciation in more diverged taxa [31]. We show that the nuclei of sperm produced by a sterile hybrid male appear paired, and hence we call them ‘needle-eye sperm’. We confirm the presence of this phenotype across a variety of interspecies crosses in *Drosophila*, suggesting that it is potentially universal within this genus. We then identify the genomic regions that are responsible for the formation of NE sperm using a bulked segregant analysis on 10th-generation backcross hybrid males formed between *Drosophila simulans* and *D. mauritiana*, and evaluate candidate genes within these regions.

## 2. Materials and methods

### 2.1. *Drosophila* maintenance, strains and crosses

Species pairs were chosen using the same criteria as in Kanippayoor *et al.* [30]. Briefly, we chose species pairs that were relatively recently diverged, produce sterile male hybrids that still make sperm, and the pairs came from different groups within the *Drosophila* genus: the sibling species *D. simulans* and *D. mauritiana* from the melanogaster group diverged ~260 000 years ago [32], *D. pseudoobscura* and *D. persimilis* from the obscura group diverged ~500 000 years ago [33], and *D. arizonae* and *D. mojavensis* from the repleta group diverged ~0.6–1.2 Mya [34,35]. The melanogaster and obscura groups diverged ~30–60 Mya; melanogaster and obscura diverged from repleta ~40–75 Mya [36,37].

All *Drosophila* lines and interspecies crosses were maintained on standard Bloomington media (Bloomington Drosophila Stock Center, Bloomington, IN, USA) and were housed at 22C on a 14 h:10 h light–dark cycle at 75% humidity unless otherwise noted. Species pairs were the same as those used in Kanippayoor *et al.* [30], for which interspecies hybrid males are sterile but produce sperm. *Drosophila mojavensis* (#1501-1352.22), *D. arizonae* (#15081-1271.00), *D. pseudoobscura* (#114011-0121.149) and *D. persimilis* (#14011-0111.49) were obtained from the Drosophila Species Stock Center (San Diego, CA, USA). Transgenic *D. simulans* and *D. mauritiana* flies, both with green fluorescent protein (GFP)-tagged protamine B (genotype: *w^+^; pBac{3xP3-EGFP, ProtB-EGFP}11B*), were obtained from J. Belote; henceforth denoted as *sim*GFP and *mau*GFP, respectively. Protamines are used in packaging DNA in sperm heads, and hence, GFP-tagged protamines enable easy visualization of sperm head morphology.

We made F_1_ hybrid males by mating female *D. mojavensis* with male *D. arizonae,* and female *D. pseudoobscura* with male *D. persimilis*, as well as the reciprocal crosses of those species pairs. Female *sim*GFP and male *mau*GFP were also mated to make hybrids, but the reciprocal cross was not performed as it produces hybrid males that do not produce sperm [38].

### 2.2. Introgression by backcrossing with phenotype-based selection

**Figure 1.**
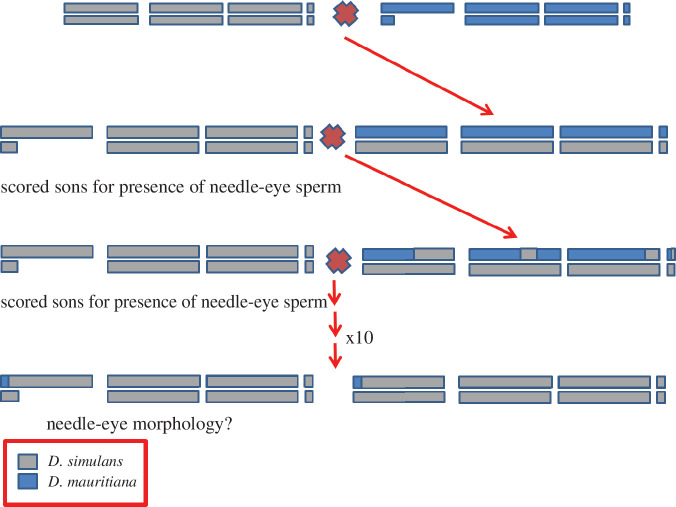
Crossing scheme for the generation of hybrid sons producing either needle-eye sperm (sterile) or motile, non-needle-eye sperm (fertile). The three major chromosome pairs are shown, with the sex chromosomes on the left. In this figure, a single F_1_ daughter, produced from a cross between *D. simulans* (female) with *D. mauritiana* (male; X chromosome is paired with a shorter chromosome, Y), was then backcrossed to a single male *D. simulans*. Sons were scored for the presence of needle-eye sperm, and their sisters were used to singly mate with a male *D. simulans*. This process was repeated for 10 generations of backcrossing. This crossing scheme was repeated for the reciprocal cross (not shown), wherein an F_1_ daughter and subsequent offspring were instead backcrossed to *D. mauritiana* for 10 generations.

To introgress (cross in) loci contributing to the male sterility phenotype between *D. simulans* and *D. mauritiana*, five vials were made combining five young (2 days old), virgin female *sim*GFP mated to five *mau*GFP males (same age). Twenty of the resulting F_1_ hybrid *sim/mau* virgin females were aged 2 days and singly mated back (i.e. first-generation backcrossed: BC1) to either one *sim*GFP male (BCS1) or one *mau*GFP male (BCM1; figure 1), 10 females per each direction of backcross. Virgin daughters and sons that were produced were separated. Males were aged for 1 day and then scored for ‘NE sperm’ or ‘fertile sperm’ (see §3 for sperm morphology description). Fertile sperm was denoted as being motile and containing a single nucleus. The first vial that was identified as producing some or all sons with NE sperm was used to make the next generation, with 10 daughters from that vial placed in single mating pairs for the next generation of backcrossing (i.e. BC2). This phenotype-based selection (NE sperm and fertile sperm) and introgression mapping were performed for 10 generations of backcrossing. In the final generation (BC10), 20 females were used in single-paired crosses to increase the number of available sons for subsequent sequencing.

### 2.3. Imaging sperm nuclei

We collected sterile hybrid males produced from the interspecific cross between *D. arizonae* and *D. mojavensis* (offspring denoted as *ari/moj*) and *D. pseudoobscura/D. persimilis* (offspring denoted as *pse/per*) upon eclosion and aged them for 1 day. Testes were placed in approximately 100 µl of Testes Buffer (183 mM KCl, 47 mM NaCl, 10 mM Tris-HCl) and immediately transferred to 20 µl of 0.5 µl/ml of 4’,6-diamidino-2-phenylindole (DAPI), ripped open to allow contents to expel and incubated for 5 mins on a siliconized coverslip. We washed the testes contents using Testes Buffer three times and then mounted the testes on a glass slide. Testes of sterile hybrid males produced from the interspecific cross between *sim*GFP and *mau*GFP (offspring denoted as *sim/mau*) were extracted using Testes Buffer and mounted on a glass slide; no DAPI was used.

Images were taken using a Leica DMI6000 B inverted microscope and were analysed using MetaMorph software (Molecular Devices, Sunnyvale, CA). Some samples were captured using Z-stacking with images deconvolved with AutoQuant Deconvolution software (AutoQuant Imaging, Inc.).

### 2.4. Sequencing and analysis

We scored males produced after 10 generations of backcrossing to *sim*GFP (BCS10) or *mau*GFP (BCM10) for either NE sperm or wild-type (WT) sperm. We pooled together 30 BCS10 males from a single surviving vial with NE sperm (BCS10NE) and 30 BCS10 males from the same single vial with WT sperm (BCS10WT) for DNA extraction. These males all had the same mother. The same procedure was done with BCM10 males, making samples BCM10NE and BCM10WT; however, these came from pooling males across five vials as there were not enough surviving males from any one vial. Therefore, BCM10 males did not have the same mother but did have the same grandmother. Only 30 males were pooled for each phenotype, as more individuals would reduce the efficacy of the DNA extraction process and overall yield. Samples were pooled based on the Mendelian nature of the causal loci (see §3) indicating that a single causal locus was likely to be identified.

DNA extraction for each phenotype followed a modified protocol of the QIAGEN Gel Extraction Kit (Qiagen, Toronto, ON, Canada). Each sample was held at 95°C for 5 min in a buffer solution (1 M Tris-HCl, 0.5 M EDTA and 5 M NaCl) containing 200 µg/ml proteinase K. Isopropanol was then added to each sample and incubated overnight at −20°C. DNA purification was performed as directed by the QIAGEN Gel Extraction Kit. Purified DNA samples were then sequenced using the Illumina HiSeq 2000 platform with paired-end 100 bp reads at Génome Québec Innovation Center (Montréal, QC, Canada) using a target insert size of 400 bp.

We assembled the FASTQ files generated by Illumina sequencing technology using two Linux-based de novo assemblers: SOAPdenovo 1.05 and its companion program, GapCloser 1.12 [39], and Abyss 1.5.2 [40] at Kmer values of 41 to 91. The assembly with the highest N50 value was chosen for further analysis. The six sample pools (BCS10NE, BCS10WT, BCM10NE, BCM10WT, pure species *sim*GFP and pure species *mau*GFP) were analysed for single nucleotide polymorphisms (SNPs) associated with the NE phenotype using population phenotype-based selection and introgression followed by whole-genome resequencing (PopPsiSeq). This method was built upon the earlier PsiSeq approach of Earley and Jones [41]. This allowed the comparison of allele frequencies between groups (e.g. replicates of a treatment), whereas earlier comparisons were on an individual basis and based on the presence or absence of a fixed variant. By working on a population level, PopPsiSeq was able to use differences in allele frequency between groups (of which fixation is an extreme case), an approach that can accommodate pooled samples. This increased statistical power and allowed the examination of polygenic traits.

Reads were preprocessed with FASTP [42] for quality control and analytics. Starting FASTQ files contained a total of 1.22G reads; after quality control, this dropped to 1.18G. Reads were first mapped to a reference genome using the BWA SAMPE/SE algorithm. Then, the alignment file was filtered for uniqueness (i.e. a read must be aligned optimally with no alternative or runner-up hits, XT:A:U.*X0:i:1.*X1:i:0), mapping/sequencing quality (-q 20 F 0x0100 F 0x0200 F 0x0300 F 0 x04) and deduplication. The filtered alignment mappings were used to jointly call variants in VCF format via FreeBayes [43] using standard filters. To build this VCF, all six samples were called jointly; however, not all sites were called in all samples (e.g. owing to coverage differences).

Once the SNPs were called, the VCF file was split into subsets. The *sim*GFP and *mau*GFP variants were treated as ancestral populations, while other subsets represented experimental treatments, such as all *simulans* backcrosses, all NE mutants or the NE *simulans* backcross. For each SNP still meeting minimum requirements (biallelic, not missing in any sample), the subgroup-wide allele frequency was calculated. Using *sim*GFP and *mau*GFP as ancestral, the distance to the *simulans* frequency and the *mauritiana* frequency were calculated for each SNP, for each subset to give an allele frequency shift relative to each ancestral population. These frequency shifts were averaged by 100 kb sliding windows. The data were re-analysed with windows of 10 kb, 100 kb, 500 kb and 1 Mb wide and with rolling SNP bins of 120, 1250 and 12 500 SNPs to confirm the robustness of the results with different window/bin parameters.

The patterns of average allele frequency shift were then analysed to identify regions that contained *D. simulans* SNPs within a genetic background of *D. mauritiana* that was uniquely found in males with NE sperm (sterile) versus no NE sperm (fertile). A reciprocal study was performed in parallel wherein regions containing *D. mauritiana* SNPs were identified within the genetic background of *D. simulans* in males with NE versus no NE sperm. We could not formulate a meaningful way to test for ‘statistical significance’ using this method of comparing shifts in allele frequencies, and thus our approach only identifies associations that produce a large visible skew in allele frequencies in association with the NE phenotype.

### 2.5. Candidate genes

We examined whether any candidate genes within the region with shifted allele frequencies on the X chromosome had strong patterns of SNP association with the NE phenotype (see §3). To identify candidate genes, we performed FlyBase (release FB2023_05) *D. melanogaster* sequence search for X:12998500..25000000, exported the results into FlyBase HitList and sorted for those with expression in male gonads. See §3 for the reason this sequence search range was chosen. Note that this search extended beyond the end of our sequencing reads (which were not robust after approximate base position 17 000 000 owing to heterochromatin effects). We identified 25 candidate genes, expressed in spermatozoon (*Lgr4*, *Bap60*, *DNAlig4*, *Fer3HCH*, *Nna1*, *CG10996*), testis (*lncRNA:CR45622*, *gce*, *mh*, *CG32820*, *CG32819*5), spermatogonium (*Dhc16F*, *CG15373*, *CG17450*, *tilB*), primary spermatocyte (*p-cup*, *pcm*, *r-cup*), male germline stem cell (*Nup153*), spermatid (*Ulp1*), seminal receptacle (*wupA*), mature primary spermatocyte (*Ste*), spermatocyte (*Ada3*), gonad (*CG15446*) or germline cell (*CG4318*) in *D. melanogaster*. Two of these genes did not have suitable correlates in the *D. simulans* sequence and were excluded (*lncRNA:CR45622*, *Ste*); there were two genes in *simulans* similar to *Ulp1*, and so we used the one (*LOC6726423*) that had the greatest homology to the *melanogaster* locus; *CG32820*, *CG32819* and *CG17450* are tandem duplicates which are all listed as orthologues of the *simulans LOC6726613*. These adjustments left us with 21 candidate loci. The 21 gene sequences were extracted from the gene annotation GTF, converted to BED format and extended by 10 kb in each direction using the slop utility of BEDtools [44]. These loci were used as intervals for averaging the PopPsiSeq frequency the way windows are for whole-genome scans. To sample the background genomic distribution, the loci of a gene list were shuffled across their containing contigs without an overlap to generate pseudoloci intervals. Ten such shuffles were generated per list. The genetic background was generated similarly, by shuffling the gene annotation and picking the first genes with the same chromosome distribution.

## 3. Results

### 3.1. Sperm head morphology and ‘needle-eye’ sperm

**Figure 2.**
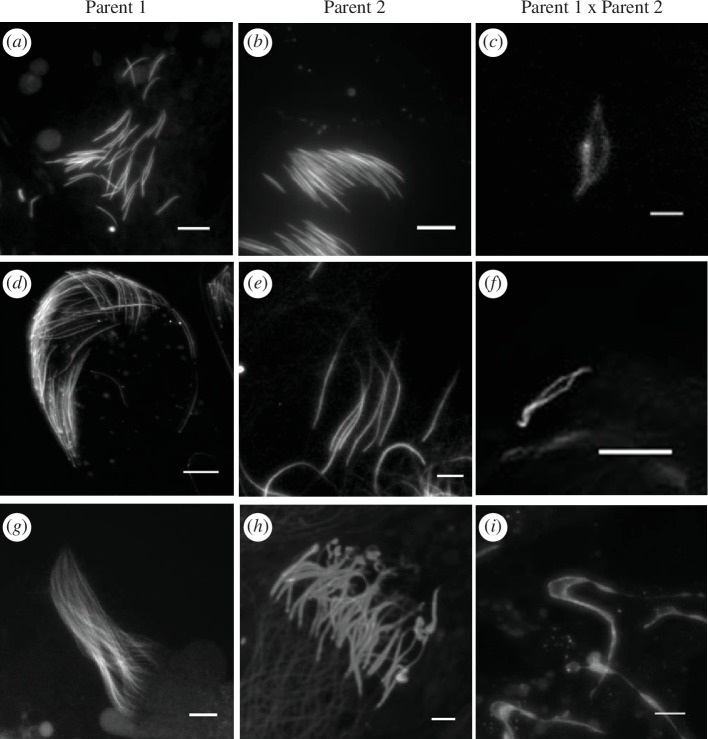
Novel ‘needle-eye’ sperm phenotype in interspecies males. Fluorescent microscopy of sperm heads in six species of *Drosophila* and their interspecific sterile hybrid males: *D. simulans* (*a*), *D. mauritiana* (*b*), *sim/mau* (*c*), *D. pseudoobscura* eusperm (long, fertilizing sperm morph) (*d*), *D. persimilis* eusperm (*e*), *pse/per* (*f*), *D. arizonae* (*g*), *D. mojavensis* (*h*) and *ari/moj* (*i*). Sperm fluoresce either owing to a GFP transgene (*a*–*c*) or owing to DAPI staining (*d*–*i*). Note that sperm heads appear as long white dashes in the images, and there are many sperm heads visible in panels *a*,*b*,*d*,*e*,*g* and *h.* Sperm nuclei of males from each parental species are properly individualized within a sperm bundle or ejaculate, whereas sperm nuclei of sterile hybrid males appear paired at polar ends of the nuclei, denoted as needle-eye sperm. Panels *c*,*f* and *i* were taken at 63× magnification, while all other panels were taken at 40× magnification. Scale bars for *c*,*f* and *i* represent 0.05 mm, while all other scale bars represent 0.1 mm.

Sperm head morphology was examined in young (<2 days old) males for six different species of *Drosophila* and their respective interspecies hybrids (figure 2). As expected, all sperm heads of each pure species (*n* = 10) possessed properly separated nuclei (figure 2*a,b,d,e,g,h*). In contrast, hybrid males (*n* = 10) produced between female *D. simulans* and male *D. mauritiana* all possessed only sperm with heads that appear attached at opposite ends of the nuclei, with a void between both heads (figure 2*c*). We describe this morphology as ‘needle-eye’ sperm. The NE sperm morphology was also present in all F_1_ hybrid males produced from the interspecific cross between *D. pseudoobscura* and *D. persimilis* (figure 2*f*), and between *D. arizonae* and *D. mojavensis* (figure 2*i*). Interestingly, while pure-species *D. pseudoobscura* and *D. persimilis* males can produce sperm of three [45] and two [46] different head and tail lengths, respectively, the sperm heads from *pse*/*per* F_1_ hybrids appeared to all be of similar size, and all displayed the NE morphology. Compared with pure species, hybrid sperm is approximately the same length (*ari/moj*), twice as long (*sim/mau*) or half as long (*pse/per*) as previously reported [30]. Sperm from sterile male hybrids were taken at 63× magnification instead of 40× magnification to ensure proper visualization of NE morphology; however, sperm from pure-species males were also observed at 63× magnification to confirm the absence of NE morphology (data not shown).

### 3.2. Prevalence of needle-eye sperm in backcross males

We scored the number of sons producing NE sperm for each mother after backcrossing to either *D. simulans* (BCS10) or *D. mauritiana* (BCM10) for 10 generations (table 1). Note that these mothers were all sisters to males who produced NE sperm in the previous generation. In the final backcross of single pairings to *D. simulans* males, the four females (out of 20) that made any sons with NE sperm produced approximately 50% BCS10 sons with NE sperm (table 1). Similarly, in the final backcross to a male *D. mauritiana*, the 15 females (out of 20) that made any NE sons produced approximately 50% BCM10 sons with NE sperm (table 1). No male produced a mix of WT and NE sperm, and all presented with a single phenotype. All other females produced sons with only WT sperm (data not shown). This suggests that after 10 generations of backcrossing, a single dominant region may be responsible for the formation of NE sperm in either BCM or BCS sterile males.

**Table 1.** Prevalence of needle-eye sperm in BCS10 and BCM10 males.

backcross	female	number of sons with needle-eye sperm/total # scored	% sons producing needle-eye sperm
BCS10	1	4/9	44%
	2	15/30	50%
	3	5/9	56%
	4	10/20	50%
BCM10	1	5/10	50%
	2	4/7	57%
	3	4/8	50%
	4	3/6	50%
	5	5/10	50%
	6	5/10	50%
	7	5/10	50%
	8	4/10	40%
	9	4/10	40%
	10	4/10	40%
	11	5/10	50%
	12	4/10	40%
	13	5/10	50%
	14	5/10	50%
	15	5/6	83%

### 3.3. Genomic mapping of regions underlying ‘needle-eye’ sperm morphology

Statistics on genome sequencing assembly are presented in table 2 (Dryad doi: 10.5061/dryad.g1jwstqvq). The amount of data generated per strain ranged from 3.1 to 15.2 Gb. Raw FASTQ files contained a total of 1.22G reads; however, after quality control (QC), this dropped to 1.18G. Assembly sizes ranged from 125 to 144 Mb resulting in genome coverage of approximately 25× to 110×. Coverage was similar across chromosome arms, with approximately half as deep of coverage on the X, as would be expected in males. The draft assemblies were fragmented with scaffold counts ranging from 44 281 to 358 157. N50 values ranged from 2 to 113 kb.

**Table 2.** Amount of sequencing data, draft assembly size, scaffold number and N50 value for sequenced *Drosophila*.

males^a^	data	assembly	scaffolds	N50
BCM10WT	3.1 Gb	125 Mb	44 281	78.7 kb
BCM10NE	3.6 Gb	126 Mb	46 755	97.2 kb
BCS10WT	4.5 Gb	144 Mb	358 157	23.3 kb
BCS10NE	4.6 Gb	134 Mb	173 476	1.9 kb
*mau*GFP	13.8 Gb	144 Mb	142 005	112.6 kb
*sim*GFP	15.2 Gb	137 Mb	171 468	23.1 kb

^a^ Males listed were produced from backcross (BC) to *D. mauritiana* (M) or *D. simulans* (S) and showed wild-type (WT) or needle-eye (NE) phenotypes or were from the parental strains (*mau*, *sim*) with fluorescently tagged (GFP) sperm.

Allele frequencies associated with the NE phenotype were assessed using PopPsiSeq. For details of sequence coverage and quality, see electronic supplementary material, tables S1–S4 and figures S1–S3. We identified 1.5–1.9M SNPs, averaging ~12 per kb (electronic supplementary material, table S5). Note that our ability to detect significant regions in the two directions of backcross may have been influenced by the different amounts of sample pooling in the groups. BCS was composed of pooled males that came from the same mother, which reduced background genetic variation in the sample but also increased the likelihood of shared non-relevant introgressed pieces of genome among the individuals in the sample. BCM was composed of pooled males from five mothers but the same grandmother, which increased background noise but reduced the chance of non-relevant introgressed genomic regions being shared among individuals within the sample.

**Figure 3.**
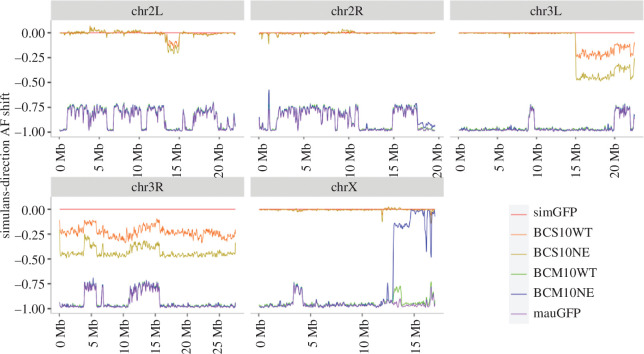
Shift in allele frequencies in backcross males with needle-eye sperm phenotype. Allele frequencies (AF) of SNPs in pure species *sim*GFP and *mau*GFP males compared with males from the 10th-generation backcross to *simulans* (BCS10) or *mauritiana* (BCM10) displaying wild-type (WT) or needle-eye (NE) sperm phenotypes. The *y*-axis is the shift in AF and not the AF itself (see electronic supplementary material, figure S4 for an explanation of allele frequency shifts). Frequencies are shown across the five main chromosome arms (X, 2L, 2R, 3L and 3R), with the *sim*GFP allele as the baseline (0). Note that *mau*GFP SNPs are –1; where they are above –1 indicates segregating or overlapping SNPs within the parental species strains. Regions where the BCS10NE SNP frequency is more shifted than WT towards *mau*GFP, or where BCM10NE is more shifted than WT towards *sim*GFP, are introgressed SNPs associated with the NE phenotype.

There was one introgressed region of modest association in BC *simulans* on the third chromosome and a separate region of very strong association on the X chromosome in BC *mauritiana* (figure 3). These results were robust to different window and SNP bin parameters (electronic supplementary material, figures S5 and S6). A region towards the centromere on 3L spanning approximately 8 Mb adjacent to the centromere (starting approximately 3L:1 50 07 000 in the *sim* genome; electronic supplementary material, figure S7) and the entirety of 3R contained *D. mauritiana* SNPs in BCS males that were associated with the NE phenotype. The association was modest along the chromosome, and no particular subregion of the third chromosome had a strong association with sterility. This chromosome did not have an association with NE sperm in BCM males.

**Figure 4.**
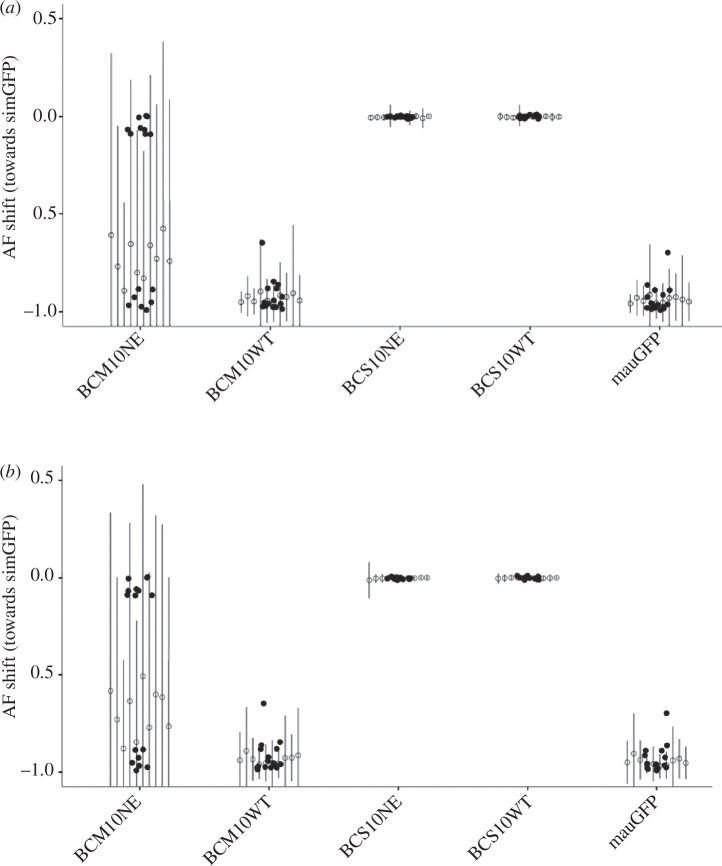
Allele frequencies of SNPs within candidate genes on the X chromosome. Average allele frequency (AF) shift for 21 candidate genes in pure species *mau*GFP and backcross (BCM10, BCS10) males with wild-type (WT) or needle-eye (NE) morphology, relative to allele frequencies present in pure-species *sim*GFP. An AF value of 0 indicates *sim*-like alleles, while a value of –1 indicates *mau*-like alleles. Solid dots represent the allele frequency for each gene, shown centred over the *x*-axis labels. The data was shuffled 10× (see §2); open dots are the mean with vertical lines representing 2s.d. on either side representing the error of the distribution of each shuffle, calculated using (*a*) X chromosome intervals of the same size as the candidate genes or (*b*) the same number of X chromosome genes as the candidate gene list. Note that BCM10NE has a large distribution owing to the bimodal nature of its variance. The three dots near 0 for BCM10NE are the genes *tilB, r-cup* and *CG15446*. Note that the slightly elevated gene in BCM10WT (*Tektin-3*) is the same gene elevated in *mau*GFP, indicating that variants in this gene are not fully fixed in *sim*GFP and/or *mau*GFP.

Conversely, a region spanning approximately 4 Mb adjacent to the centromere on the X chromosome (starting approximately X:1 29 99 000 in the *sim* genome; electronic supplementary material, figure S8) was strongly associated with NE sperm in BCM males but not BCS males (figure 3). We further explored 21 candidate genes that have a function related to male fertility (see §2) that were found within this strongly associated region. The candidate genes were assessed for *simulans*-like SNP allele frequencies in BCM10NE males, indicating that the locus was introgressed from *simulans* (figure 4). Only three candidate genes displayed *simulans*-like SNP allele frequencies: *touch insensitive larva B* (*tilB*), *ryder cup* (*r-cup*) and *CG15446*. Another candidate factor could be the ribosomal DNA (rDNA) repeats involved in X–Y pairing [47], but owing to the repeat nature of these rDNA sequences, we were unable to assess whether these specific sequences were introgressed (see §4).

## 4. Discussion

Here, we uncovered a previously uncharacterized sperm phenotype for sterile hybrid males and found that it is present across multiple interspecies pairs spanning the genus *Drosophila*. The phylogenetically widespread occurrence of the unusual NE sperm morphology in sterile hybrid *Drosophila* males adds to the increasing evidence of a consistent point of spermatogenic failure in hybrid males, which has been found so far to occur in Meiosis I in species pairs spanning the genus *Drosophila* [30], two species of *Saccharomyces* yeast [48], two species of mosquito (*Anopheles gambia* complex [49]) and two subspecies of mouse (*Mus musculus* [50]), suggesting that there may be an underlying, universal mechanism for the evolution of hybrid male sterility. Additional tests in ZW species are needed to determine if this is a male-specific trend or applicable to hybrid sterility in heterogametic females as well.

When we selected the NE phenotype, we were surprised to discover that the genetic basis of this F_1_ sterility phenotype appears to be dominant and Mendelian, with ~50% of male offspring having NE sperm, even after 10 generations of backcrossing. Genetic mapping of sterility revealed a weak association spanning most of the third chromosome for NE morphology in backcross *simulans* males. While this potentially may be resolved with a greater sample size, it is plausible that our results were instead owing to the sterility phenotype in this backcross resulting from multiple different loci, any one of which is sufficient alone to induce sterility, confounding our power to detect them using pooled samples.

We found a very strong association with the centromeric region of the X chromosome and NE morphology in backcross *mauritiana* males, with no other region of the genome showing association in these males. When we assessed the potential causal factors within this region, we identified three candidate genes associated with the NE phenotype: *touch insensitive larva B* (*tilB*), *ryder cup* (*r-cup*) and *CG15446*. Very little is known about *CG15446*; other than that it is transcribed in male gonads (FlyBase). The *r-cup* gene is one of the few loci post-meiotically transcribed during spermatogenesis. Its transcripts subcellularly localize to the tips of sperm tails in elongating spermatid bundles [51], suggesting a potential role in sperm motility. Lastly, *tilB* encodes a protein required for cilium function, affecting sperm motility when disrupted [52].

A more intriguing potential causal factor within this region of the X chromosome is a small region of the genome that is necessary for heteromorphic chromosome pairing. While homomorphic chromosomes rely on shared overall homology during meiotic pairing, heteromorphic chromosomes depend on alternative mechanisms for pairing. In *Drosophila*, intergenic 240 bp rDNA repeat regions found within the X heterochromatin and Y centromere are required for X–Y pairing [47]. This region is rapidly evolving and differs among even closely related species of *Drosophila* [53]; it is plausible that the molecular machinery involved in X–Y pairing would be similarly rapidly evolving. Furthermore, combining X and Y chromosomes from different species has been shown to result in sterility, regardless of the autosomal complement (e.g. between *D. simulans* and *D. mauritiana*: [54,55], indicating that some aspect of heteromorphic chromosomes themselves may be responsible for F_1_ sterility. Likewise, in mice, the pseudoautosomal region involved in pairing X and Y chromosomes is strongly associated with hybrid sterility when X and Y are from different species [56]. However, interspecies *Drosophila* females bearing two X chromosomes from one species and Y chromosome from another are fertile [54]. It has long been proposed that the difference in these effects is potentially owing to spermatogenesis being particularly susceptible to disruption, but this does not explain the observation of Haldane’s rule in ZW females. How can these results be reconciled?

We propose that F_1_ hybrid sterility results from components that are uniquely expressed in heterogametic individuals *because* they are heterogametic; specifically, the molecular components involved in the coordination of heteromorphic chromosomes during meiosis must be complementary. In *Drosophila*, females would not need to express the machinery involved in heteromorphic chromosome pairing and separation, and thus a non-complementary interaction between these molecules would not arise within an XXY interspecies female. We further propose that hybrid sterility arises owing to failures of proper segregation rather than failure to pair. Indeed, there appears to be a consistent pattern across a number of species for hybrid sterility failures arising in Meiosis I owing to failure in separation [30,48–50], and our observation here of the consistent NE phenotype further supports that heteromorphic interspecies sterility is affected by failures in chromosome separation during meiosis.

One limitation of this study is that short-read sequencing data usually do not allow proper assembly of long repeated sequences. We, therefore, could not directly assess if the 240 bp repeat sequence involved in X–Y pairing was associated with the NE phenotype; this warrants further exploration. We currently know remarkably little about the molecules (and associated genes) involved in pairing and separating heteromorphic sex chromosomes during meiosis and how they may diverge across closely related lineages. The possibility that divergence in the machinery involved in sex chromosome alignment and separation may be broadly responsible for Haldane’s rule adds motivation for investigating the molecular basis of this process.

## Data Availability

All data are presented in the manuscript except genome sequence data which are uploaded to Dryad [57]. Supplementary material is available online [58].
